# First evaluation of PET-based human biodistribution and radiation dosimetry of ^11^C-BU99008, a tracer for imaging the imidazoline_2_ binding site

**DOI:** 10.1186/s13550-018-0429-x

**Published:** 2018-07-30

**Authors:** Ashwin V. Venkataraman, Nicholas Keat, James F. Myers, Samuel Turton, Inge Mick, Roger N. Gunn, Eugenii A. Rabiner, Jan Passchier, Christine A. Parker, Robin J. Tyacke, David J. Nutt

**Affiliations:** 1 Neuropsychopharmacology Unit, Centre for Psychiatry, Division of Brain Sciences, Imperial College London, 5th Floor Burlington Danes Building, Hammersmith Hospital campus, 160 Du Cane Road, London, W12 0NN UK; 2 Imanova Limited, Imperial College London, Hammersmith Hospital, Burlington Danes Building, Du Cane Road, London, W12 0NN UK; 3 Restorative Neurosciences, Imperial College London, Burlington Danes Building, Hammersmith Hospital campus, 160 Du Cane Road, London, W12 0NN UK; 4 Experimental Medicine Imaging, GlaxoSmithKline Research & Development Limited, Gunnels Wood Road, Stevenage, SG1 2NY UK

**Keywords:** Biodistribution, Dosimetry, Positron emission tomography, ^11^C-BU99008, Imidazoline2

## Abstract

**Background:**

We measured whole body distribution of ^11^C-BU99008, a new PET biomarker for non-invasive identification of the imidazoline_2_ binding site. The purpose of this phase I study was to evaluate the biodistribution and radiation dosimetry of ^11^C-BU99008 in healthy human subjects.

**Methods:**

A single bolus injection of ^11^C-BU99008 (296 ± 10.5 MBq) was administered to four healthy subjects who underwent whole-body PET/CT over 120 min from the cranial vertex to the mid-thigh. Volumes of interest were drawn around visually identifiable source organs to generate time-activity curves (TAC). Residence times were determined from time-activity curves. Absorbed doses to individual organs and the whole body effective dose were calculated using OLINDA/EXM 1.1 for each subject.

**Results:**

The highest measured activity concentration was in the kidney and spleen. The longest residence time was in the muscle at 0.100 ± 0.023 h, followed by the liver at 0.067 ± 0.015 h and lungs at 0.052 ± 0.010 h. The highest mean organ absorbed dose was within the heart wall (0.028 ± 0.002 mGy/MBq), followed by the kidneys (0.026 ± 0.005 mGy/MBq). The critical organ was the heart wall. The total mean effective dose averaged over subjects was estimated to be 0.0056 ± 0.0004 mSv/MBq for an injection of ^11^C-BU99008.

**Conclusions:**

The biodistribution of ^11^C-BU99008 has been shown here for the first time in humans. Our dosimetry data showed the total mean effective dose over all subjects was 0.0056 ± 0.0004 mSv/MBq, which would result in a total effective dose of 1.96 mSv for a typical injection of 350 MBq of ^11^C-BU99008. The effective dose is not appreciably different from those obtained with other ^11^C tracers.

## Background

The imidazoline_2_ binding site (I_2_BS) is thought to be located on the mitochondrial membranes of astrocytes [1]. Changes in post-mortem binding density of the I_2_BS has implicated them in a range of psychiatric conditions such as depression and addiction, along with neurodegenerative disorders such as Alzheimer’s disease (AD) and Huntington’s chorea [2]. Preclinical models have also demonstrated functional interactions with the opioid system, where I_2_BS ligands have been shown to reduce tolerance to morphine [3] and alleviate some elements of the morphine withdrawal syndrome in rats [4]. Recently, some I_2_BS ligands have been shown to have nociceptive and analgesic effects in different models of pain [5–7], and a novel I_2_BS ligand is currently undergoing phase II clinical trials as a novel treatment for neuropathic pain and acute non-specific pain states.

The location of I_2_BS on glial cells and the possibility that they may in some way regulate glial fibrillary acidic protein [8] have led to increased interest into the role of I_2_BS and I_2_BS ligands in conditions characterised by marked gliosis. The density of I_2_BS has been shown to increase in AD postmortem [2], and it has also been suggested that I_2_BS may be a marker for the severity and malignancy of human glioblastomas [9]. The fact that I_2_BS is increased in postmortem AD brains and that they are located on astrocytes mean that a radioligand that binds to the I_2_BS may prove to be a very useful research tool for understanding both the role of I_2_BS and the astrocytic arm of the neuroinflammatory process in AD [10–15].

^11^C-BU99008 (2-(4,5-Dihydro-1H-imidazol-2-yl)-1-[11C]methyl-1H-indole / 2-(4,5-Dihydro-1H-imidazol-2-yl)-1-methyl-1H-indole) has been extensively characterised in pre-clinical species and demonstrated to be a suitable research tool for the quantification of brain I_2_BS availability (rat [16], pig [17], and rhesus [18]). We have also shown ^11^C-BU99008 binds with a significantly lower affinity to monoamine oxidase type B (MAO_B_) and is selective for I_2_BS in these preclinical species. ^11^C-BU99008 is currently being validated as a radiotracer for I_2_BS in healthy human brain [19-21].

The safety and tolerability of ^11^C-BU99008 PET imaging have been investigated in healthy volunteers. A previous investigation of the distribution of this PET radioligand was performed in healthy rhesus monkeys in order to determine a safe dose of radiation to human subjects following administration of ^11^C-BU99008 (unpublished data). No data are yet available on the biodistribution and radiation safety of ^11^C-BU99008 in humans. The aim of the present phase I study was to determine this data using PET imaging of healthy volunteers using radiodosimetry.

## Methods

### Subjects

Biodistribution data were obtained from four healthy subjects (four men; mean age 51 years; range 45–55 years) who underwent whole-body ^11^C-BU99008 PET/CT (Imanova Ltd., London) and were used for dosimetry analysis. Informed consent was obtained from all individual participants included in the study. Suitability for participation included the absence of clinically significant illness or disease, which was assessed by interview, physical examination, electrocardiogram, vital signs measurements, routine blood tests, urine drug screen and alcohol breathalyser. The protocol was approved by West London Research Ethics Committee (IRAS number 14/LO/1741), ARSAC (630/3764/32214), and listed as a phase 1 study (ClinicalTrials.gov Identifier: NCT02323217). The mean and standard deviation of the administered mass was 1.675 ± 0.629 μg (range, 0.84–2.17 μg). The target activity was 300 MBq, with a mean administered value of 296.1 ± 10.5 MBq (range, 281.8–306.8 MBq); no adjustment was made for subject weight. The mean and standard deviation specific activity was 40.9 ± 20.6 GBq/μmol (range, 25.9–70.4 GBq/μmol). There were no adverse or clinically detectable pharmacologic effects in any of the four subjects. No significant changes in vital signs were observed.

### Radiopharmaceutical preparation

^11^C-BU99008 was prepared by N-alkylation of the precursor BU99007 using ^11^C CH_3_I as previously described [17, 18]. BU99007 (1.0 mg) was dissolved in dimethylformamide (300 μL) in a 1-mL glass vial, and tetrabutylammonium hydroxide (20 μL of a 0.1 M methanol solution) was added. ^11^C CH_3_I was delivered to the vial at room temperature in a helium carrier gas stream. After ^11^C CH_3_I delivery, the vessel was heated at 40 °C for 2 min. At the end of the labelling, the reaction mixture was injected onto a semipreparative HPLC column (Eclipse SB-phenyl column [Agilent]; 250 × 9.4 mm). HPLC purification was performed with a mobile phase of acetonitrile and ammonium formate (50 mM; pH 9.9; 50:50) at a flow rate of 10 mL/min. The product fraction (retention time, ~ 5.5 min) was collected and diluted with 100 mL of water. This solution was passed through a C18 Sep-Pak (Waters), rinsed with water (10 mL), and eluted off with ethanol (2 mL), followed by saline (8 mL). The analysis of chemical and radiochemical purity was performed by analytical HPLC (Eclipse SB-phenyl column; 150 × 4.6 mm) using a mobile phase of acetonitrile and ammonium formate (50 mM; pH 9.9; 50:50) at a flow rate of 2 mL/min. To confirm the radiopharmaceutical identity, a sample of the purified material was also co-injected with a non-radiolabelled sample of BU99008. Two ^11^C-BU99008 productions were analysed using different HPLC conditions (Eclipse XDB-C18 column [Agilent]; 150 × 4.6 mm) (32% acetonitrile: 68% ammonium formate [50 mM; pH 9]; 1.5 mL/min) to confirm that the product was chemically and radiochemically pure.

### ^11^C-BU99008 PET/CT image acquisition

^11^C-BU99008 was administered intravenously. Imaging was performed using one of two whole-body PET/CT scanners (Siemens Biograph 6 True Point and HiRez). Before each emission imaging session, a whole-body low-dose CT scan was acquired for attenuation correction (130 kV, 15 mAs). PET emission data were acquired in four male subjects for a total of approximately 120 min after injection, proceeding from the cranial vertex to mid-thigh (6–7 bed positions per scan, depending upon subject size). Subsequent whole-body static scans with durations of 1, 2, 3, 5, and 5 min per bed position were acquired over this period to produce data with scan-start times at approximately 0, 10, 25, 45, 80 and 115 min post-injection. Due to a differing number of beds, the time for each scan was similar but not identical for each subject. The acquired data were iteratively reconstructed with corrections for attenuation, scatter, and randoms. The reconstruction protocol employed 2D OSEM with four iterations and 16 subsets, as well as a three-dimensional Gaussian filter with a full width at half maximum of 5 mm.

### Activity quantification and dose calculation

PET and CT image data were imported to MRIcroN [22] and volumes of interest drawn using the combination of PET scan and/or CT that most clearly depicted organs relevant to radiation dosimetry (full list given in Table 2). Measured activity concentrations were trapezoidally integrated over all five scans, with the activity in the final scan assumed to decay with no further redistribution. The integrated activity concentrations per unit injected activity were multiplied by OLINDA/EXM 1.1 organ volume to derive organ residence times (equivalent time that unit activity spends in that organ per injected unit activity). These values were used as source organs for input to OLINDA/EXM 1.1 using both the mean residence times over all subjects, as well as for each subject individually. The residence times over all organs were added, and this value was subtracted from the total residence time for carbon-11 (0.489 h) to calculate the ‘remainder’ organ residence time. OLINDA produced organ absorbed doses, organ effective dose contributions and total effective doses for each subject.

## Results

**Fig. 1:**
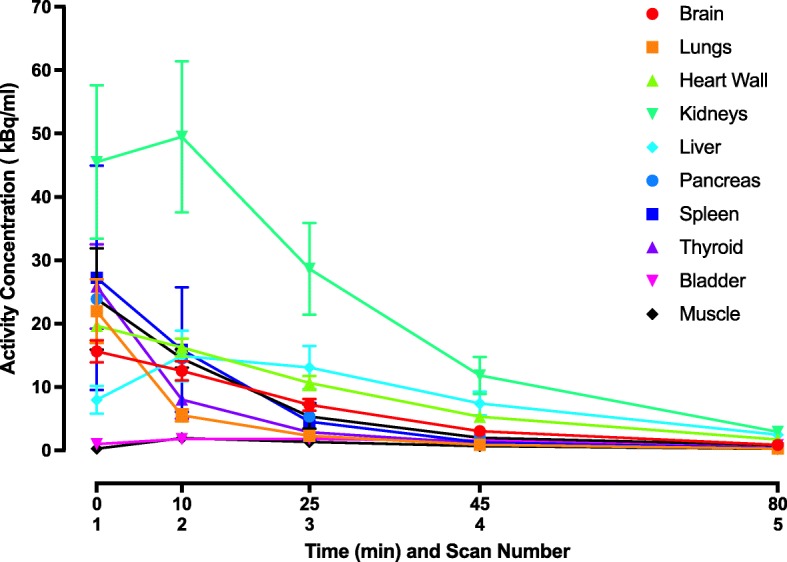
Mean time-activity curves in select organs (subjects 1–4). Bars indicate standard deviation. Time refers to approximate start time of scan after injection of ^11^C-BU99008

**Fig. 2:**
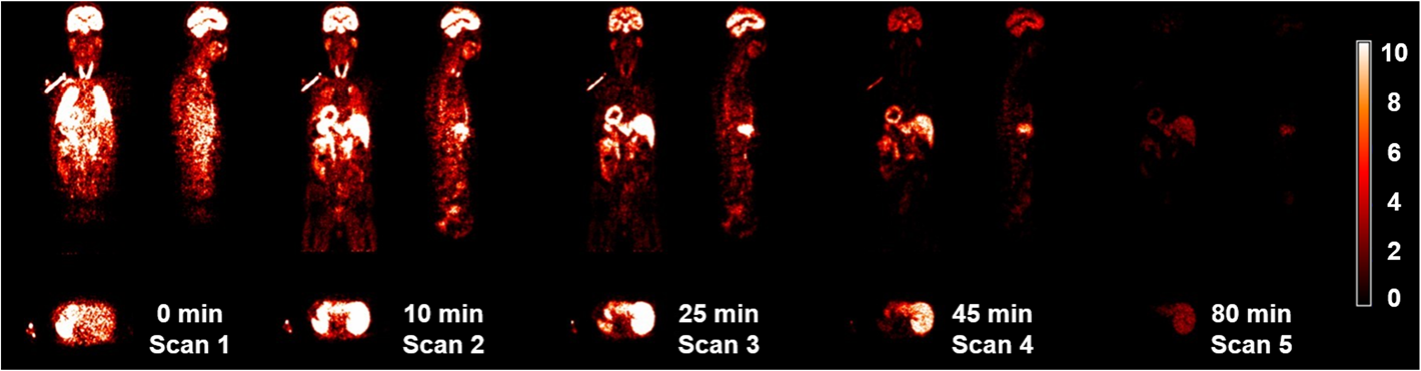
Whole-body PET images of a representative subject at scans 1–5 after injection of ^11^C-BU99008. Coronal, axial and sagittal sections are shown. Time refers to approximate start time of scan after injection of ^11^C-BU99008

The characteristics of the subjects are documented in Table 1. Injection of ^11^C-BU99008 was well tolerated, and no pharmacological effects were observed. Figure 1 shows the mean time activity curves in organs of interest across all subjects. Figure 2 shows a representative coronal, axial and sagittal whole-body PET ^11^C-BU99008 biodistribution in a male subject during scans 1–5 after injection. During scans 1–2, uptake is visualised mainly in the kidneys, spleen and heart. During scans 3–5, uptake is mainly visualised in the kidneys, heart and liver. The highest uptake was in the kidneys, followed by the spleen. The liver shows slower uptake that is retained to a greater extent than other organs. In contrast with many other radioligands, low activity concentrations were seen in the urinary bladder.

**Table 1:** Patient characteristics

Characteristic	Data
Sex (*n*)	
Male	4
Female	0
Age (years)	
Mean	50.8
Range	45–55
Body weight (kg)	
Mean	82.2
Range	67.6–110.8
Injected dose (MBq)	
Mean ± SD	296.1 ± 10.5
Range	281.8–306.8
Injected mass (μg)	
Mean ± SD	1.675 ± 0.629
Range	0.84–2.17

The mean administered activity to the four participants was 296 MBq (range 281–306 MBq). Mean residence times in source organs are displayed in Table 2. These values reflect both the activity concentration in the tissues of interest, as well as their volume. The longest residence time (± standard deviation) was in muscle at 0.100 ± 0.023 h, followed by the liver at 0.067 ± 0.015 h and lungs at 0.052 ± 0.010 h. The mean residence time in the brain was 0.035 ± 0.004 indicating high brain uptake. The mean total residence time was 0.37 ± 0.0504 h, leaving the residence time for activity injected but not measured within the assessed organs (the ‘remainder’ organ) as 0.114 ± 0.050 h.

**Table 2:** Residence times of ^11^C-BU99008 in organs

Source organ	Mean residence time (h)	Standard deviation (h)
Adrenals	0.000	0.000
Brain	0.035	0.004
Breasts	0.000	0.000
Gallbladder	0.001	0.000
LLI	0.002	0.001
Small intestine	0.010	0.004
Stomach	0.011	0.003
ULI	0.002	0.001
Heart contents	0.012	0.002
Heart wall	0.027	0.002
Kidneys	0.027	0.006
Liver	0.067	0.018
Lungs	0.052	0.011
Muscle	0.100	0.027
Ovaries	N/A	N/A
Pancreas	0.002	0.000
Red marrow	0.003	0.001
Cortical bone	0.014	0.005
Trabecular bone	0.001	0.000
Spleen	0.005	0.003
Testes	0.000	0.000
Thymus	0.000	0.000
Thyroid	0.000	0.000
Urinary bladder	0.002	0.000
Uterus	N/A	N/A
Total	0.374	0.058
Remainder	0.114	0.058

Data are hours (mean ± SD, *n* = 4)

The mean absorbed doses in measured target organs are shown in Table 3. The highest mean absorbed dose (and the critical organ) was in the heart (0.028 ± 0.002 mGy/MBq), followed by the kidneys (0.026 ± 0.005 mGy/MBq) and lungs (0.015 ± 0.002 mGy/MBq).

**Table 3:** Effective doses in measured target organs

Target organ	Mean absorbed dose (mGy/MBq)	Standard deviation (mGy/MBq)
Adrenals	0.008	0.003
Brain	0.008	0.001
Breasts^a^	0.002	0.000
Gallbladder wall	0.006	0.001
LLI wall	0.003	0.000
Small intestine	0.005	0.001
Stomach wall	0.008	0.001
ULI wall	0.004	0.000
Heart wall	0.028	0.002
Kidneys	0.026	0.005
Liver	0.013	0.003
Lungs	0.015	0.002
Muscle	0.002	0.000
Ovaries^b^	0.002	0.000
Pancreas	0.009	0.001
Red marrow	0.002	0.000
Osteogenic cells	0.003	0.001
Skin	0.001	0.000
Spleen	0.009	0.004
Testes	0.001	0.000
Thymus	0.003	0.000
Thyroid	0.006	0.001
Urinary bladder wall	0.002	0.000
Uterus^b^	0.002	0.000
Total body	0.003	0.000

Data are mGy/MBq (mean ± SD, *n* = 4)

^a^Doses are for male breast only, and may be different for female breasts

^b^Doses to uterus and ovaries are based on doses from other source organs only

The total mean effective dose over all subjects was 0.0056 ± 0.0004 mSv/MBq, which would result in a total effective dose of 1.96 mSv for a typical injection of 350 MBq of ^11^C-BU99008.

## Discussion

This study is the first to determine the biodistribution and dosimetry of ^11^C-BU99008 in human subjects. No adverse reactions or clinical changes were observed. The estimated absorbed doses to critical and radiation-sensitive organs are acceptable and considered to be compatible with serial scans in a single research subject, according to the International Commission on Radiological Protection [23], risk category IIb, with doses of less than 10 mSv. The estimated radiation doses are consistent with those for other neuroreceptor ligands labelled with carbon-11 [24].

One limitation of this study is that given all subjects were male—a residence time for ovaries and uterus were not determined and absorbed doses to these organs are based on irradiation by other source organs only. Similarly, residence times and doses for breast are for male breast only and may be potentially different for female breasts.

The urinary bladder wall is often reported in dosimetry studies as being an organ with a particularly high absorbed dose, despite carbon-11 having a short half-life. ^11^C-BU99008 did not accumulate in the bladder, but did in the kidneys. The liver also showed slower uptake that is retained to a greater extent than other organs. Pre-clinical literature shows specific binding in both the kidney and liver [25]. This could suggest that the tracer takes longer to reach the bladder as it is not just filtered rapidly by the liver and kidney but may have an additional element of specific binding.

Of note, there is accumulation of radioactivity in the heart wall and pancreas. There is no significant binding in the adrenals, despite this possibility based on pre-clinical literature [25]. Imidazoline binding sites, type 3, have been reported in the pancreas [26, 27] and are thought to be involved in insulin secretion. The finding in the heart wall was novel; as to our knowledge, I_2_BS have not previously been reported in the heart. Further research is needed to clarify this unexpected finding.

## Conclusion

The biodistribution and internal dosimetry profiles for ^11^C-BU99008 in humans indicate a favourable radiation risk profile, hence making the use of whole-body ^11^C-BU99008 PET/CT feasible for evaluating the I_2_BS and safe for consecutive studies when clinically required.
